# Granulomatosis with polyangiitis with pyoderma gangrenosum, acne, hidradenitis suppurativa-like features

**DOI:** 10.1016/j.jdcr.2024.05.040

**Published:** 2024-06-26

**Authors:** Rebeka Dejenie, Melinda Wong, Nicholas Love, Yong He, Maxwell A. Fung, Danielle M. Tartar

**Affiliations:** a School of Medicine, University of California, Davis, Sacramento, California; b Department of Dermatology, University of California, Davis, Sacramento, California

**Keywords:** granulomatosis with polyangiitis, pyoderma gangrenosum, pyoderma gangrenosum, acne, hidradenitis suppurativa (HS), and psoriatic arthritis (PAPASH), pyoderma gangrenosum spectrum disorders

## Introduction

Granulomatosis with polyangiitis (GPA) is an uncommon necrotizing granulomatous vasculitis involving small and medium-sized vessels, most commonly affecting the skin, respiratory tract, and kidney.^1^ Rarely, GPA can present with necrotizing ulcers, making it difficult to discern GPA from other ulcerating skin disorders including pyoderma gangrenosum (PG) and other neutrophilic dermatoses.^2^ There is scant literature on a patient with simultaneous GPA and PG, acne, hidradenitis suppurativa (HS) (PAPASH, and psoriatic arthritis) presenting symptoms.^3^^,^^4^ Here, we present a case of a 16-year-old patient who initially presented with chronic necrotizing lesions who was ultimately diagnosed with GPA with PAPASH-like features. He showed rapid improvement with corticosteroids, methotrexate, and rituximab.

## Case report

A 16-year-old male with a long-standing history of acne vulgaris and joint pains, worse in the shoulders and sternum, initially presented with inflammatory nodules in his left axilla and left ear. He was given a working diagnosis of HS and was treated with oral doxycycline before transitioning to adalimumab (Humira) 40 mg weekly after failing to show improvement with doxycycline. Two months after initiation of Humira, he developed scleritis, iritis, epistaxis, and lower extremity purpura. Cytoplasmic-antineutrophil cytoplasmic antibodies (C-ANCA) and antihistone antibodies were positive. Humira was held, but symptoms persisted.

**Fig 1:**
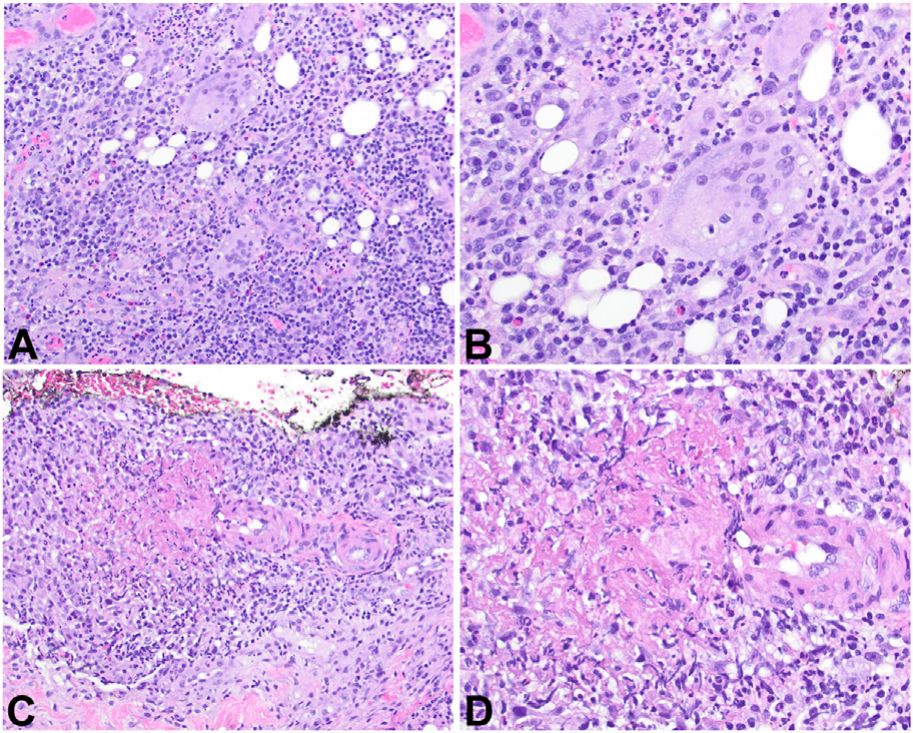
Biopsy of the left axilla (**A**: 20×; **B**: 40×) and left upper arm (**C**: 20×; **D**: 40×) reveal suppurative granulomatous dermatitis with focal vasculitis.

**Fig 2:**
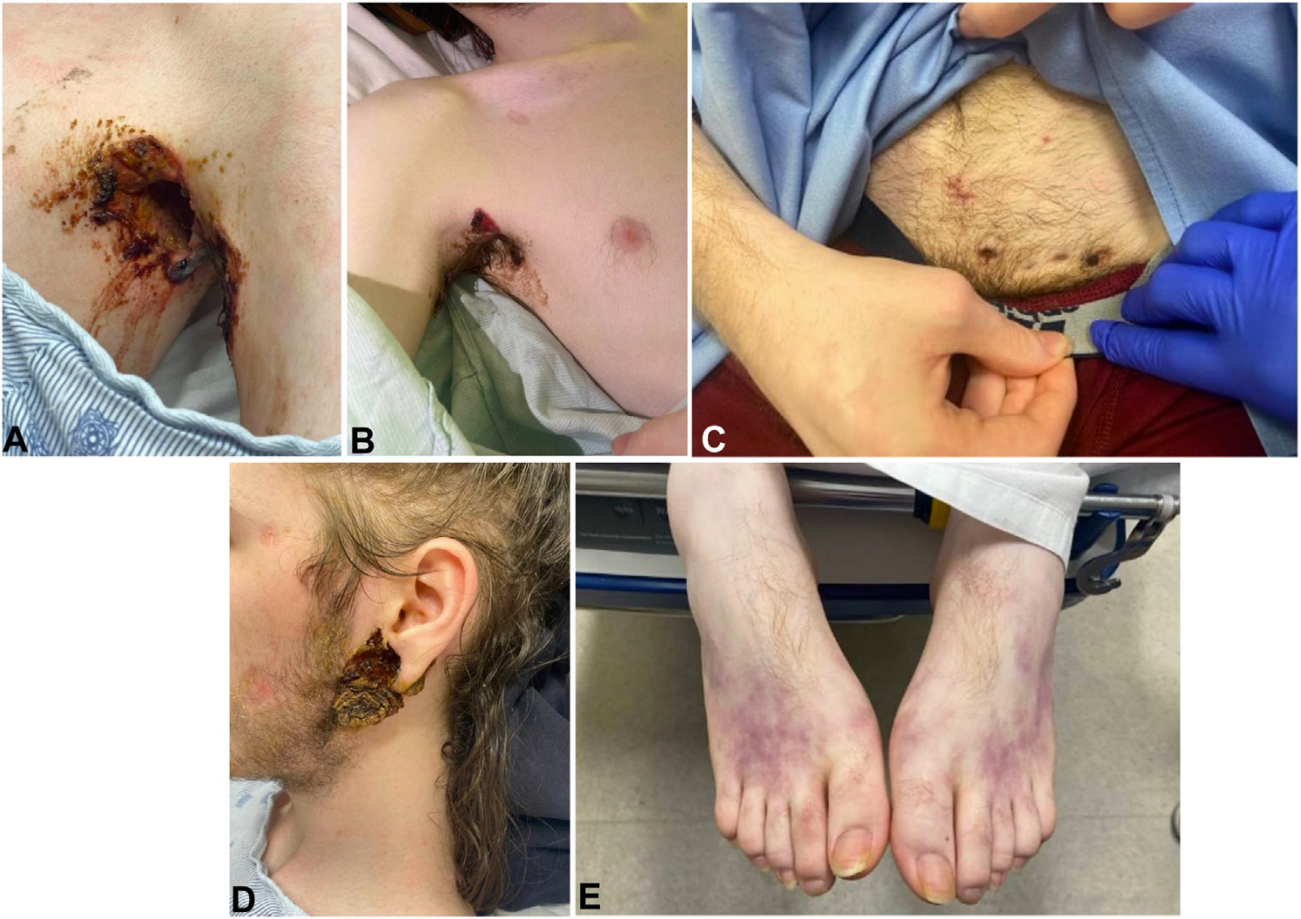
Chronic ulceronecrotic erosive lesions of the left axilla (**A**), right axilla (**B**), abdomen (**C**), left auricle (**D**), and ill-defined mottling of the bilateral lower extremities (**E**).

**Fig 3:**
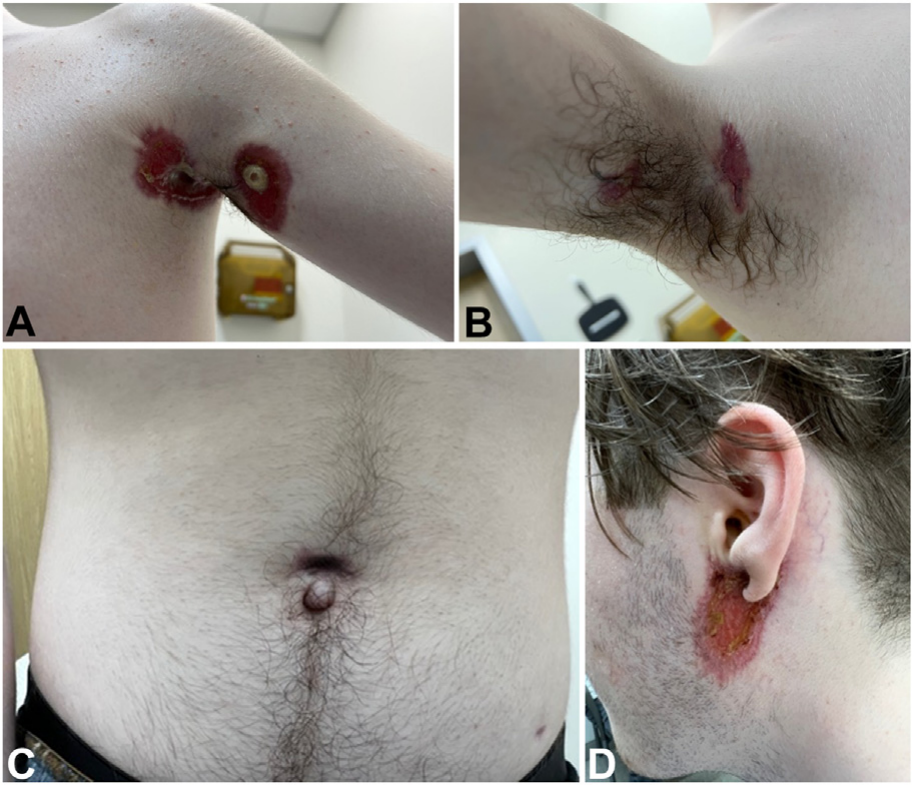
Left axilla (**A**), right axilla (**B**), abdomen (**C**), left auricle (**D**) at follow-up after initiation of immunosuppressive regimen.

Given the severity of his symptoms, a biopsy of the left axilla was performed, which revealed suppurative granulomatous dermatitis with focal vasculitis (Fig 1, Fig 2, Fig 3). The patient was started on prednisone 60 mg daily and referred to our institution. As vasculitis is not often a feature of HS, this was thought to be an early clue of a more widespread vasculitis.

Patient was admitted to the hospital given rapid progression of his multifocal ulceronecrotic erosive lesions involving bilateral axilla, left auricle, abdomen, and groin. Axillary ulcerations were noted to have significant undermining (Fig 2, *A* and *B*). Inflammatory papules, double-headed comedones and inflammatory nodules were noted on the abdomen (Fig 2, *C*). The ulceronecrotic lesion on the left auricle and peri-auricular neck had eroded through his left lobule (Fig 2, *D*). The patient was also noted to have a retiform purpura of the bilateral lower extremities (Fig 2, *E*). He also endorsed widespread joint pain and general fatigue and malaise.

The patient endorsed mild epistaxis but denied shortness of breath or cough. Given the history of C-ANCA positivity, chest and sinus computed tomography were ordered, revealing a 1.7 cm cavitating nodular lesion in the right upper lung. Acid-fast bacteria stains (×3) were negative (×3) from sputum and QuantiFERON gold serology testing excluded *Mycobacterium tuberculosis*. Computed tomography of the face revealed extensive sinus disease involving the right maxillary, ethmoid, and frontal sinuses with significantly less inflammatory disease involving the left-sided sinuses. Urinalysis revealed moderate occult blood but no protein. Creatine was within normal limits. Kidney biopsy was obtained, which revealed focal segmental necrotizing (pauci-immune) glomerulonephritis as well as patchy acute tubular injury and patchy interstitial inflammation, compatible with GPA. Given his widespread joint pain, X-Ray of the bilateral sternum and shoulders was obtained, but was negative for damage secondary to inflammatory arthropathy. Ophthalmology was consulted during his admission and found no evidence of current uveitis.

Given this constellation of findings, a diagnosis of GPA with PAPASH-like features was favored. Given his widespread ulceronecrotic lesions, the patient was initially treated with broad spectrum IV antibiotics (vancomycin for 3 days, levofloxacin for 7 days, metronidazole for 7 days). Tissue and blood cultures ultimately grew Group A streptococcus. Once cultures were negative, the patient was started on pulse dose (500 mg daily × 3 days) methylprednisolone and was started on oral doxycycline for both anti-inflammatory properties and prevention of future secondary infection. Prednisone 60 mg daily was resumed following methylprednisolone pulse.

Multi-disciplinary discussions between dermatology, nephrology, pulmonology, and rheumatology were held and the patient was ultimately treated with methotrexate 25 mg subcutaneously weekly (with leucovorin 5 mg weekly 24 hours after methotrexate), and rituximab 1 g/kg ×2 doses given 2 wk apart. He was also started on sulfamethoxazole-trimethoprim 400-80 mg tablets twice daily on weekends for pneumocystis prophylaxis. Prednisone was slowly tapered by 5 mg/wk.

Two weeks after discharge, the patient showed significant improvement with decreased pain, increased mobility, and decreased inflammation in the axillae, abdomen, and auricle (Fig 3, *A-D*). The patient was maintained on weekly methotrexate. C-ANCA levels normalized with treatment and are now undetectable. At the time of diagnosis, C-ANCA was 16.1 at time of diagnosis, 7.3 2 weeks after initiation of therapy, and was undetectable by month 5 of therapy. Genetic testing for PAPASH-associated genes through the Autoinflammatory and Autoimmune panel from Invitae laboratories was negative, including PSTPIP1, further supporting the PAPASH-like features as a manifestation of GPA and not a primary diagnosis.

## Discussion

This case highlights an unusual presentation of GPA in a pediatric patient that was initially diagnosed with hidradenitis suppurativa. The diagnosis of GPA was supported by the C-ANCA positivity, cutaneous biopsy findings, cavitary lesions in the sinuses and lung, as well as necrotizing glomerulonephritis. The patient’s long-standing history of acne, the HS-like lesions on the abdomen, the PG-like lesions in the axillae and auricles, and his widespread joint pain are consistent with a PAPASH-like presentation. Our report demonstrates how GPA is time sensitive diagnosis given its potential for rapid progression; delays in treatment results in poor patient outcomes. Indeed, previous studies have shown that patients with untreated GPA have up to a 90% 2-year mortality.^5^^,^^6^ This unique presentation of GPA with ulceronecrotic lesions can be difficult to discern from other skin disorders and requires a high clinical suspicion. Isolated PG in association with C-ANCA positivity is well-known, but PAPASH-like presentations are rare. There have been two prior reports of ANCA-associated PAPASH-like syndromes, one associated with P-ANCA (kawa) and one associated with C-ANCA (ursani). Table I summarizes all prior reports of ANCA positivity associated with arthritis, acne, or HS. Recognizing GPA despite overlapping syndromes (in this case, overlap with PAPASH features) is important as initiating prompt treatment improves patient outcomes. In this case, the patient had secondary bacteremia, which is a concern with ulceronecrotic lesions, especially in the setting of his recent immunosuppression. Thus, surveillance for infection is mandatory.

**Table I:** Summary of previously reported ANCA – associated PAPASH syndromes

ANCA positivity	PAPASH-like features	Sex	PSTPIP1	Treatment	Reference
P-ANCA	Arthritis, Acne, HS, PG	Male	Negative	Prednisolone, Minocycline, Dapsone	Kawanishi et al^7^
C-ANCA	Arthritis, Acne, HS, PG	Male	Unknown	Prednisone	Ursani et al^8^
C-ANCA	Acne, HS, PG	Male	Unknown	Prednisolone, Cyclophosphamide	Singh et al^3^
C-ANCA	HS, Arthritis	Male	Unknown	Prednisone, Methylprenisolone, Cyclophosphamide, Rituximab, Doxycycline	Alavi et al^4^
C-ANCA	HS	Female	Unknown	Methylprednisolone, Plasmapheresis, cyclophosphamide, Azathioprine, Methotrexate, Rituximab	Alavi et al^4^

*HS*, Hidradenitis suppurativa; *PG*, pyoderma gangrenosum.

## Conflicts of interest

None dislosed.
